# Determinants of orthodontic treatment acceptance among university students in Northern China: a structural equation modeling approach

**DOI:** 10.3389/fpubh.2026.1806852

**Published:** 2026-03-24

**Authors:** Peng Zhang, Zhiliang Chu, Jiarui Li, Yue Chai, Renbin Han, Li Dong

**Affiliations:** 1 School of Stomatology, Jinzhou Medical University, Jinzhou, Liaoning, China; 2 School of Health Management, Jinzhou Medical University, Jinzhou, Liaoning, China; 3 Innovation and Entrepreneurship Center, Jinzhou Medical University, Jinzhou, Liaoning, China

**Keywords:** China, orthodontics, service quality, structural equation modeling, treatment acceptance, university students

## Abstract

**Background:**

Malocclusion represents a prevalent oral health concern with notable psychosocial implications. Despite high treatment need among young adults in China, the transition from awareness to actual uptake of orthodontic care remains inadequately understood, particularly in student populations.

**Objective:**

This study aimed to assess the willingness to undergo orthodontic treatment and identify its predictors among university students in Liaoning Province, China.

**Methods:**

A cross-sectional survey was conducted using multistage stratified cluster random sampling across 14 cities in Liaoning Province. A total of 1,299 students aware of orthodontic treatment completed a structured questionnaire. Data were analyzed using *t*-tests, ANOVA, and structural equation modeling (SEM) to examine the relationships between service quality dimensions (reliability, responsiveness, assurance, empathy), social norms, satisfaction, and treatment acceptance.

**Results:**

No significant demographic differences were found in relation to acceptance willingness or its predictors (all *p* > 0.05). SEM indicated that reliability (β = 0.172), responsiveness (β = 0.135), assurance (β = 0.230), and empathy (β = 0.135) positively influenced satisfaction. Satisfaction (β = 0.179), along with reliability (β = 0.185), responsiveness (β = 0.199), assurance (β = 0.212), empathy (β = 0.147), and social norms (β = 0.106), directly enhanced willingness to accept treatment.

**Conclusion:**

Perceived service quality, social norms, and satisfaction are pivotal in shaping orthodontic acceptance among Chinese university students. Enhancing reliability, responsiveness, assurance, and empathy in clinical practice may improve treatment uptake in this population.

## 1 Introduction

Malocclusion represents a significant global public health challenge, adversely affecting oral function, facial aesthetics, and psychosocial wellbeing (1). Its high prevalence among adolescents and young adults underscores its societal impact, with national surveys in China reporting rates exceeding 67% in the relevant age groups (2). The university student population, situated at a critical juncture of personal and social development, exhibits a particularly pronounced need for orthodontic care, driven by both functional requirements and aesthetic concerns (3). However, a substantial gap persists between this evident need and the actual uptake of treatment, suggesting that factors beyond clinical necessity govern the decision-making process (4). Investigating the determinants that shape the willingness to undergo orthodontic treatment among university students is therefore not merely an academic inquiry but a pressing public health priority. Understanding these factors is essential for designing effective interventions, optimizing healthcare resource allocation, and ultimately improving the oral health-related quality of life for this large demographic (5). To accurately assess perceptions of service quality, social norms, and anticipated satisfaction—all of which require a foundational understanding of orthodontic care—this study specifically targeted students with prior awareness of orthodontic treatment. This criterion ensures that respondents could provide meaningful evaluations of the hypothetical service attributes presented in the questionnaire, thereby enhancing the validity of the measurement model.

Extensive research has been dedicated to unraveling the factors influencing orthodontic treatment-seeking behavior. Globally, studies have identified key determinants including the perceived severity of malocclusion, aesthetic motivation, cost considerations, anticipated pain and treatment duration, and the influence of family and peers (6–10). Theoretical frameworks such as the Health Belief Model and the Theory of Planned Behavior have been successfully applied to structure this inquiry, highlighting constructs like perceived benefits, barriers, and subjective norms (11, 12). Within China, researchers have begun to explore cultural and socio-economic influences on orthodontic acceptance (13, 14). These collective efforts have significantly advanced our understanding, demonstrating that treatment acceptance is a multi-factorial phenomenon. However, notable gaps in the literature remain. Firstly, much of the existing research focuses on adolescents under parental guidance or adult patients already in care, providing limited insight into the decision-making autonomy of university students—a group characterized by transitional independence and specific financial constraints (15, 16). Secondly, prior studies often examine influencing factors in isolation using bivariate analyses, failing to capture the complex, interdependent relationships between multidimensional constructs such as service quality perceptions, social norms, satisfaction, and behavioral intention (17, 18). Thirdly, there is a paucity of research focusing on specific regions within China, particularly Northeast China, where economic and cultural contexts may differ from the more commonly studied coastal metropolises (19, 20). Consequently, while previous work has effectively cataloged potential influences, the integrated mechanisms through which these factors collectively shape pre-treatment willingness in a regionally specific, non-clinical student sample are not yet fully understood.

To address these complexities and overcome the limitations of traditional analytical methods, Structural Equation Modeling (SEM) has emerged as a powerful statistical technique in behavioral and health services research (21). SEM enables the simultaneous testing of a network of hypothesized relationships among multiple latent and observed variables, making it exceptionally suitable for examining both direct and indirect effects within a unified model (22). This capability is crucial for investigating mediating pathways—for example, how different dimensions of perceived service quality indirectly influence behavioral willingness through the mediating variable of anticipated satisfaction (23). Although SEM has been employed in dental research to model patient satisfaction and adherence (24, 25), its application to predict the willingness to accept orthodontic treatment prior to clinical engagement in a general student population is novel. This methodological approach offers a refined lens to dissect the “black box” of decision-making, allowing for a holistic test of an integrated theoretical model that combines insights from service quality management, social psychology, and health behavior theories (26, 27). By adopting SEM, our study employs a contemporary and robust analytical strategy with the potential to clarify the nuanced relationships that previous, less integrated methods may have obscured (28–30).

Guided by this rationale, the present study was conducted to systematically investigate the determinants of orthodontic treatment acceptance among university students in Liaoning Province, Northeast China. We executed a large-scale, cross-sectional survey utilizing a multi-stage stratified cluster random sampling method across 14 cities. A sample of 1,299 students with prior awareness of orthodontics completed a structured questionnaire assessing demographics, perceptions of service quality (reliability, responsiveness, assurance, empathy), social norms, anticipated treatment satisfaction, and willingness to accept treatment. After preliminary analyses using *t*-tests and ANOVA to examine demographic variations, we constructed and validated a comprehensive structural equation model. This model explicitly hypothesized and tested the direct effects of service quality dimensions and social norms on acceptance willingness, as well as the mediating role of satisfaction between service quality and the final outcome. Our work contributes to the field by providing the first SEM-based, regionally representative analysis of orthodontic acceptance drivers among Chinese university students. It moves beyond listing factors to modeling their interrelations, offering a mechanistic understanding of how perceptions translate into intention. Ultimately, the findings of this study aim to provide a robust theoretical and empirical foundation for the design of targeted health communication strategies, patient-centered service improvements, and effective public health interventions aimed at bridging the orthodontic treatment gap in this important population.

## 2 Materials and methods

### 2.1 Study design and participants

A cross-sectional survey was conducted to investigate the determinants of orthodontic treatment acceptance among university students in Liaoning Province, China. The target population comprised full-time undergraduate and postgraduate students who possessed prior awareness of orthodontic treatment. To ensure the sample was representative of the provincial student population across its 14 prefecture-level cities, a multistage stratified cluster random sampling design was implemented (19).

### 2.2 Sampling procedure

The sampling was executed in three sequential stages to enhance representativeness and operational feasibility. In the first stage, stratified sampling was employed. All 14 cities were stratified into five primary strata based on the total number of universities and the size of the student population, utilizing official provincial educational statistics. Given that the cities of Shenyang and Dalian collectively account for over 70% of the province's university student population, they were each designated as independent strata. The remaining 12 cities were grouped into three strata based on geographic proximity and comparable student population sizes. The second stage involved the selection of universities using Probability Proportional to Size (PPS) sampling. From the five strata established in the first stage, 30 universities were systematically selected. The Yates-Grundy method was applied to ensure that the probability of a university being selected was strictly proportional to its student enrollment size, thereby providing each student in the province with an approximately equal chance of their institution being chosen. In the third and final stage, cluster random sampling was used to select individual participants. Within each of the 30 selected universities, one natural class was randomly chosen from each faculty or department. From each selected class, one student was invited to participate in the survey. This approach facilitated efficient data collection while maintaining the randomness of the sample.

### 2.3 Inclusion and exclusion criteria

Eligible participants were required to meet the following inclusion criteria: (1) being aged 18 years or older; (2) current enrollment as a full-time student at a university in Liaoning Province; (3) self-reported prior awareness or basic knowledge of orthodontic treatment; (4) possessing the general literacy skills necessary to independently read and comprehend the questionnaire; and (5) providing voluntary written informed consent prior to participation. Participants were excluded if they: (1) were not current university students; (2) had known cognitive impairments or severe psychiatric conditions that could compromise their ability to understand or voluntarily complete the survey; or (3) were unwilling to participate for any other reason.

### 2.4 Data collection and measures

Data were collected using a structured, self-administered questionnaire. A total of 1,400 questionnaires were distributed. After excluding incomplete or inconsistent responses, 1,299 valid questionnaires were retained for analysis, yielding a valid response rate of 92.79%. The final sample consisted of 670 female students (51.58%) and 629 male students (48.42%). The questionnaire was divided into two main sections. The first section gathered sociodemographic information, including age, gender, academic year, major field of study, monthly living expenses, household registration status (hukou), and city of residence. The second section contained the measurement scales for the study's core constructs. All items in this section were scored on a standard 5-point Likert scale, where 1 represented “Strongly Disagree,” 2 “Disagree,” 3 “Neutral,” 4 “Agree,” and 5 “Strongly Agree.” The constructs measured were adapted from established frameworks. Perceived service quality was assessed using a 13-item scale based on the SERVQUAL model, covering four dimensions: reliability, responsiveness, assurance, and empathy (27). Social norms were measured with a 3-item scale adapted from the Theory of Planned Behavior (12). Anticipated satisfaction with treatment was evaluated using a 3-item scale informed by previous patient satisfaction research (18). Finally, the dependent variable, willingness to accept orthodontic treatment, was measured by a 3-item scale adapted from health behavior studies (7, 16).

### 2.5 Statistical analysis

All statistical analyses were performed using SPSS version 27.0 (IBM Corp., Armonk, NY, United States) and AMOS version 27.0 software (IBM Corp., Armonk, NY, United States). Descriptive statistics, including frequencies, percentages, means, and standard deviations, were calculated to summarize the participants' characteristics. Independent samples *t*-tests and one-way Analysis of Variance (ANOVA) were conducted to examine potential differences in the key study variables—service quality dimensions, social norms, anticipated satisfaction, and willingness to accept treatment—across various demographic groups. A *p*-value of less than 0.05 was considered statistically significant for these tests.

To test the hypothesized relationships among the latent constructs, Structural Equation Modeling (SEM) was employed, which allows for the simultaneous analysis of complex variable relationships while accounting for measurement error (21, 22). The analysis followed a two-ste*p* approach (34). First, a Confirmatory Factor Analysis (CFA) was performed to evaluate the measurement model, assessing the validity and reliability of the latent constructs. Model fit was judged using multiple indices, including the ratio of chi-square to degrees of freedom (χ^2^/df), the Comparative Fit Index (CFI), the Tucker-Lewis Index (TLI), and the Root Mean Square Error of Approximation (RMSEA), with commonly accepted thresholds indicating good fit (28, 35).

Subsequently, the structural model was tested. This model specified the hypothesized paths: from the four service quality dimensions to anticipated satisfaction, and from anticipated satisfaction, the four service quality dimensions, and social norms directly to willingness to accept treatment. The significance of the standardized path coefficients (β) was evaluated. Paths with a *p*-value < 0.05 were deemed statistically significant, while those with *p* < 0.001 were considered highly significant. Nonsignificant paths were indicated by *p* > 0.05. Bootstra*p* procedures (5,000 samples) were used to estimate the stability of the path coefficients and to examine indirect effects (36).

## 3 Results

### 3.1 Participant characteristics

A total of 1,299 university students completed the survey and were included in the final analysis. The sociodemographic characteristics of the participants are summarized in Table 1. The sample comprised 670 females (51.58%) and 629 males (48.42%). The mean age of the participants was 20.8 years (SD = 1.9), with the majority being undergraduates across all academic years. Participants represented a diverse range of academic majors, with monthly living expenses distributed across different levels. Most participants held an urban household registration (hukou), and the sample was drawn from all 14 cities in Liaoning Province, with the largest proportions from Shenyang and Dalian, reflecting the underlying population distribution.

**Table 1:** Sociodemographic characteristics of the surveyed university students.

**Variable**	**Category**	**Frequency (*n*)**	**Percentage (%)**
Gender	Female	670	51.6
	Male	629	48.4
Age (years)	18–20	699	53.8
	21–23	472	36.3
	24–26	112	8.6
	≥27	16	1.2
Academic year	Junior	399	30.7
	Sophomore	324	24.9
	Senior	282	21.7
	Freshman	216	16.6
	Master's or above	78	6.0
Major	Medical sciences	626	48.2
	Science & engineering	348	26.8
	Humanities & social sci.	160	12.3
	Others	113	8.7
	Arts	52	4.0
Monthly living expenses (CNY)	≤ 2,000	537	41.3
	2,001–3,000	424	32.6
	3,001–4,000	172	13.2
	>4,000	166	12.8
Household registration (Hukou)	Urban	694	53.4
	Rural	605	46.6
Region of origin	Northern China	850	65.4
	Southern China	449	34.6

Total sample size *N* = 1,299.

### 3.2 Descriptive statistics and demographic comparisons

Descriptive statistics for the key study variables—the four service quality dimensions (Reliability, Responsiveness, Assurance, Empathy), Social Norms, Anticipated Satisfaction, and Willingness to Accept Treatment—are presented in Table 2. The mean scores and standard deviations for each variable are provided. The relationships between the seven demographic variables (gender, age, academic year, major, monthly living expenses, household registration, and region) and the seven key study dimensions were examined using independent samples *t*-tests and one-way Analysis of Variance (ANOVA). The results revealed no statistically significant differences (all *p* > 0.05) across any of the demographic subgroups for any of the study variables. Specifically, no significant differences were found between males and females, between students from urban and rural households, or across different regions within the province. While Assurance showed a trend toward a difference across age groups (*p* = 0.070), it did not reach the conventional threshold for statistical significance. This consistent lack of significant association indicates a high degree of homogeneity in perceptions of service quality, social norms, anticipated satisfaction, and willingness to accept orthodontic treatment within this university student population, irrespective of their basic demographic backgrounds.

**Table 2:** Analysis of sociodemographic characteristics in relation to willingness to accept orthodontic treatment and its influencing factors (x ± s).

**Variable**	**Category**	**Reliability**	**Responsiveness**	**Assurance**	**Empathy**	**Social norms**	**Satisfaction**	**Willingness**
Gender	Male (*n* = 629)	3.32 ± 0.82	3.35 ± 0.84	3.74 ± 0.84	3.47 ± 0.98	3.38 ± 1.01	3.67 ± 0.90	3.53 ± 0.82
	Female (*n* = 670)	3.33 ± 0.82	3.28 ± 0.91	3.74 ± 0.89	3.50 ± 1.01	3.38 ± 0.99	3.67 ± 0.93	3.48 ± 0.90
	*t*	−0.395	1.472	−0.025	−0.613	0.092	0.009	0.972
	*p*	0.693	0.141	0.980	0.540	0.927	0.993	0.331
Household registration	Rural (*n* = 605)	3.32 ± 0.84	3.34 ± 0.87	3.73 ± 0.85	3.52 ± 0.98	3.41 ± 1.00	3.69 ± 0.88	3.54 ± 0.82
	Urban (*n* = 694)	3.33 ± 0.80	3.29 ± 0.88	3.75 ± 0.88	3.46 ± 1.01	3.35 ± 1.00	3.66 ± 0.95	3.47 ± 0.89
	*t*	−0.114	1.077	−0.415	1.142	1.120	0.685	1.571
	*p*	0.909	0.282	0.678	0.254	0.263	0.494	0.116
Region	Northern (*n* = 850)	3.32 ± 0.82	3.32 ± 0.88	3.72 ± 0.87	3.50 ± 1.00	3.39 ± 1.00	3.66 ± 0.92	3.52 ± 0.83
	Southern (*n* = 449)	3.33 ± 0.82	3.30 ± 0.87	3.77 ± 0.86	3.46 ± 0.99	3.36 ± 1.00	3.69 ± 0.93	3.48 ± 0.90
	*t*	−0.246	0.323	−0.999	0.745	0.563	−0.578	0.674
	*p*	0.805	0.747	0.318	0.456	0.574	0.564	0.500
Age (years)	18–20 (*n* = 699)	3.30 ± 0.81	3.30 ± 0.88	3.68 ± 0.89	3.50 ± 1.00	3.35 ± 1.01	3.64 ± 0.94	3.49 ± 0.89
	21–23 (*n* = 472)	3.34 ± 0.85	3.32 ± 0.87	3.82 ± 0.84	3.50 ± 0.99	3.38 ± 0.98	3.69 ± 0.90	3.52 ± 0.84
	24–26 (*n* = 112)	3.38 ± 0.79	3.33 ± 0.88	3.74 ± 0.76	3.36 ± 0.97	3.59 ± 0.93	3.80 ± 0.83	3.59 ± 0.75
	≥27 (*n* = 16)	3.37 ± 0.80	3.27 ± 0.89	3.67 ± 0.94	3.67 ± 1.09	3.31 ± 1.28	3.60 ± 1.03	3.31 ± 0.97
	F	0.430	0.077	2.354	0.832	1.862	1.136	0.748
	p	0.731	0.973	0.070	0.476	0.134	0.333	0.523
Academic year	Freshman (*n* = 216)	3.36 ± 0.85	3.30 ± 0.86	3.69 ± 0.82	3.51 ± 1.01	3.40 ± 1.01	3.66 ± 0.92	3.59 ± 0.78
	Sophomore (*n* = 324)	3.31 ± 0.82	3.29 ± 0.88	3.74 ± 0.91	3.52 ± 0.97	3.38 ± 0.99	3.65 ± 0.99	3.51 ± 0.88
	Junior (*n* = 399)	3.31 ± 0.81	3.30 ± 0.88	3.75 ± 0.85	3.49 ± 1.01	3.39 ± 0.99	3.72 ± 0.89	3.42 ± 0.87
	Senior (*n* = 282)	3.33 ± 0.81	3.35 ± 0.89	3.77 ± 0.89	3.44 ± 1.01	3.30 ± 1.01	3.62 ± 0.90	3.56 ± 0.86
	Master's + (*n* = 78)	3.34 ± 0.79	3.36 ± 0.88	3.67 ± 0.78	3.47 ± 0.94	3.62 ± 0.97	3.78 ± 0.85	3.50 ± 0.86
	F	0.164	0.240	0.423	0.254	1.625	0.871	1.797
	p	0.957	0.916	0.792	0.907	0.166	0.481	0.127
Major	Medical (*n* = 626)	3.31 ± 0.80	3.31 ± 0.88	3.73 ± 0.90	3.47 ± 1.01	3.35 ± 0.99	3.69 ± 0.92	3.49 ± 0.89
	Sci. & eng. (*n* = 348)	3.30 ± 0.84	3.26 ± 0.90	3.75 ± 0.84	3.52 ± 0.99	3.35 ± 0.99	3.65 ± 0.91	3.52 ± 0.83
	Humanities (*n* = 160)	3.47 ± 0.79	3.35 ± 0.81	3.76 ± 0.78	3.56 ± 0.97	3.43 ± 0.98	3.70 ± 0.89	3.50 ± 0.79
	Arts (*n* = 52)	3.21 ± 0.77	3.27 ± 0.91	3.83 ± 0.96	3.47 ± 1.01	3.39 ± 1.04	3.51 ± 1.07	3.54 ± 0.86
	Others (*n* = 113)	3.33 ± 0.89	3.43 ± 0.88	3.65 ± 0.82	3.41 ± 0.96	3.56 ± 1.09	3.70 ± 0.91	3.56 ± 0.85
	F	1.556	0.882	0.490	0.531	1.188	0.574	0.245
	p	0.184	0.474	0.743	0.713	0.314	0.681	0.912
Monthly Expenses (CNY)	≤ 2,000 (*n* = 537)	3.30 ± 0.81	3.30 ± 0.86	3.69 ± 0.84	3.52 ± 0.97	3.35 ± 1.01	3.65 ± 0.90	3.48 ± 0.86
	2,001–3,000 (*n* = 424)	3.37 ± 0.85	3.30 ± 0.90	3.78 ± 0.88	3.48 ± 1.02	3.37 ± 0.99	3.69 ± 0.93	3.53 ± 0.86
	3,001–4,000 (*n* = 172)	3.31 ± 0.80	3.35 ± 0.87	3.81 ± 0.87	3.46 ± 1.03	3.41 ± 1.04	3.73 ± 0.91	3.53 ± 0.84
	>4,000 (*n* = 166)	3.30 ± 0.78	3.34 ± 0.90	3.69 ± 0.89	3.43 ± 0.97	3.48 ± 0.98	3.64 ± 0.97	3.50 ± 0.90
	F	0.587	0.224	1.577	0.383	0.752	0.497	0.362
	p	0.624	0.880	0.193	0.765	0.521	0.684	0.780

All values for Reliability, responsiveness, assurance, empathy, social norms, satisfaction, and willingness are presented as mean ± standard deviation. Group comparisons were conducted using independent samples *t*-tests for dichotomous variables (Gender, Household Registration, Region) and one-way ANOVA for variables with more than two categories (Age, Academic Year, Major, Monthly Expenses).

### 3.3 Structural equation modeling

#### 3.3.1 Measurement model assessment

**Figure 1:**
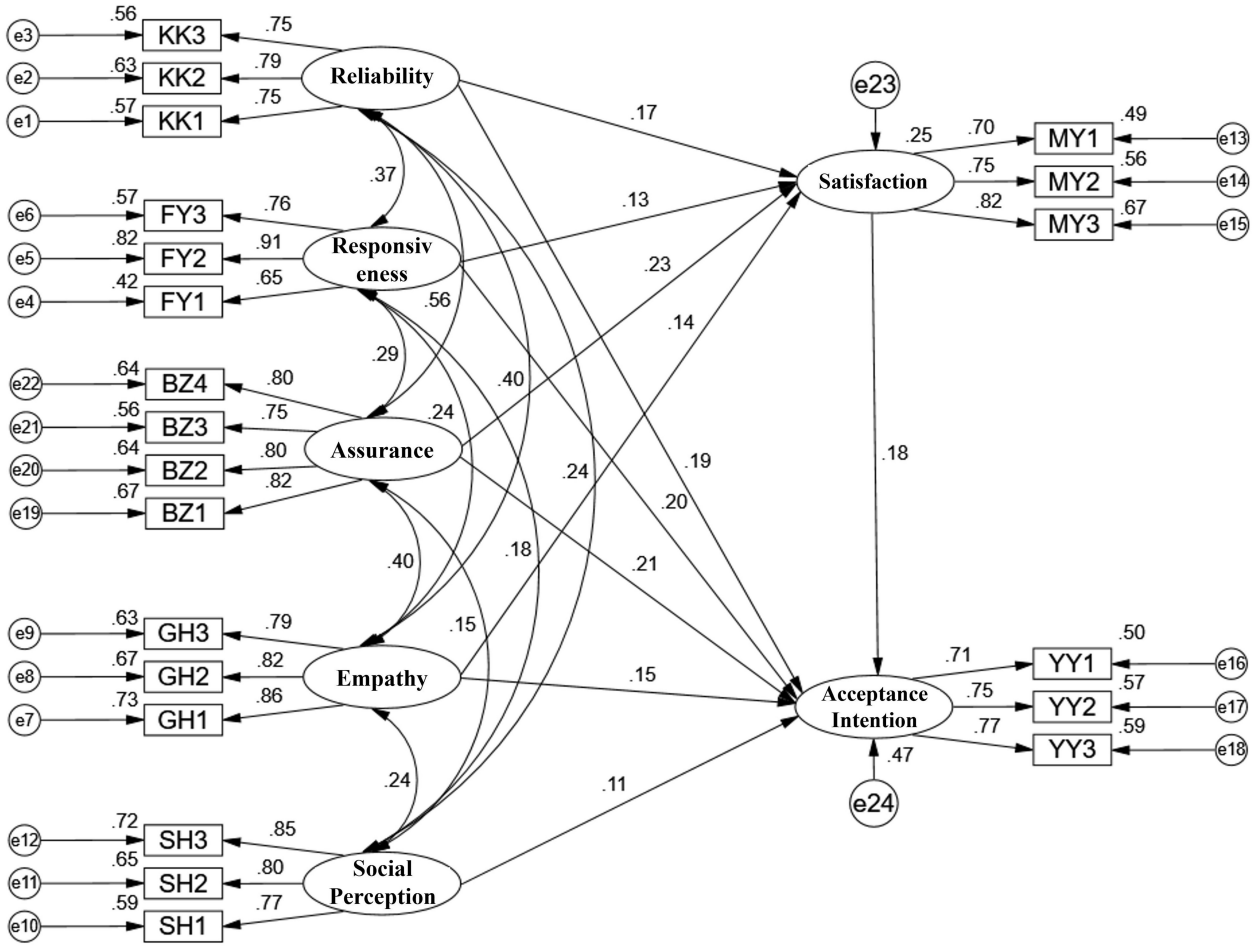
Structural equation model for the surveyed university students. Ovals represent latent constructs, and rectangles represent observed variables (questionnaire items). Single-headed arrows indicate hypothesized directional relationships, with standardized path coefficients (β) shown. All displayed paths are statistically significant at *p* < 0.001. For clarity, error terms are not shown. The observed variables correspond to the following questionnaire items: KK1, orthodontic treatment can help patients solve dental problems; KK2, orthodontic treatment can provide the promised services on time; KK3, orthodontic appointments are punctual with accurate queuing, and promises are kept; FY1, the orthodontic service provides accurate information about treatment methods, procedures, and duration; FY2, orthodontic services provide immediate and appropriate assistance; FY3, orthodontists are willing to help patients; BZ1, patients feel a sense of safety during orthodontic visits; BZ2, orthodontic service staff are friendly and courteous; BZ3, orthodontic service staff have sufficient knowledge to answer patients‘ questions; BZ4, the orthodontic consultation process is clear, well-defined, and convenient; GH1, the service provides individual attention to different patients; GH2, online consultations are available, and appointment times meet patients' needs; GH3, the service demonstrates care for individual patients; SH1, having straight teeth is a sign of being well-educated; SH2, paying attention to oral health is a marker of modern living; SN3 (SH3): having straight teeth leaves a good impression on others; MY1, overall, I have a high evaluation of orthodontic treatment; MY2, i quite appreciate current orthodontic services; MY3, I believe current orthodontic services can meet expected outcomes; YY1, i would recommend orthodontic treatment to those in need; YY2, i would bring family members to receive orthodontic treatment in the future; YY3, when encountering orthodontic problems, I would seek help from relevant medical personnel.

Prior to testing the structural relationships, the measurement model was evaluated using Confirmatory Factor Analysis (CFA). The model included seven latent constructs: the four service quality dimensions (each with their respective observed items), Social Norms, Anticipated Satisfaction, and Willingness to Accept Treatment. The fit indices for the final measurement model demonstrated an acceptable fit to the data: χ^2^/df = 4.32, RMSEA = 0.068 (90% CI: 0.062, 0.074), CFI = 0.93, TLI = 0.91, IFI = 0.93. These values meet or exceed commonly recommended thresholds for good model fit, indicating that the hypothesized factor structure was supported by the data. The standardized factor loadings for all items on their respective latent constructs were statistically significant (*p* < 0.001) and ranged from 0.65 to 0.89, demonstrating satisfactory convergent validity. The structural equation model with standardized path coefficients is depicted in Figure 1, and the detailed model fit indices are presented in Table 3.

**Table 3:** Model fit indices for the structural equation model.

**Fit Index**	**Value obtained**	**Recommended criterion**	**Meets criterion?**
Normed chi-square (*χ^2^*/df)	3.940	< 5.00	Yes
Goodness-of-fit index (GFI)	0.951	>0.80	Yes
Adjusted goodness-of-fit index (AGFI)	0.935	>0.80	Yes
Root mean square error of approximation (RMSEA)	0.048	< 0.08	Yes
Incremental fit index (IFI)	0.958	>0.80	Yes
Tucker-lewis index (TLI)	0.948	>0.80	Yes
Comparative fit index (CFI)	0.958	>0.80	Yes

χ^2^/df, Normed Chi-square (ratio of chi-square to degrees of freedom); GFI, Goodness-of-Fit Index; AGF, Adjusted Goodness-of-Fit Index; RMSEA, Root Mean Square Error of Approximation; IFI, Incremental Fit Index; TLI, Tucker-Lewis Index; CFI, Comparative Fit Index.

#### 3.3.2 Structural model and path analysis

The structural model, specifying the hypothesized paths among the latent constructs, was tested next. The standardized path coefficients (β) and their statistical significance are detailed in Table 4. The results of the path analysis are as follows: all four dimensions of perceived service quality exerted a direct and statistically significant positive influence on Anticipated Satisfaction: reliability (β = 0.172, *p* < 0.001), Responsiveness (β = 0.135, *p* = 0.003), Assurance (β = 0.230, *p* < 0.001), and Empathy (β = 0.135, *p* = 0.004). Regarding the direct predictors of Willingness to Accept Treatment, multiple significant pathways were identified. Anticipated Satisfaction had a significant direct positive effect on Willingness (β = 0.179, *p* < 0.001). Furthermore, three service quality dimensions also demonstrated significant direct effects on Willingness, independent of Satisfaction: reliability (β = 0.185, *p* < 0.001), Responsiveness (β = 0.199, *p* < 0.001), and Assurance (β = 0.212, *p* < 0.001). The direct effect of Empathy on Willingness was also positive and significant (β = 0.147, *p* = 0.001). Finally, Social Norms had a smaller but statistically significant direct positive effect on Willingness to Accept Treatment (β = 0.106, *p* = 0.011). No other hypothesized paths were significant.

**Table 4:** Standardized path coefficients and significance tests for the structural equation model.

**Path relationship**	**β**	**S.E**.	***t*-value**	***p*-value**
Reliability → satisfaction	0.172	0.046	3.959	< 0.001
Responsiveness → satisfaction	0.135	0.038	3.995	< 0.001
Assurance → satisfaction	0.230	0.037	5.700	< 0.001
Empathy → satisfaction	0.135	0.028	3.826	< 0.001
Reliability → willingness to accept	0.185	0.041	4.632	< 0.001
Responsiveness → willingness to accept	0.199	0.034	6.285	< 0.001
Assurance → willingness to accept	0.212	0.034	5.552	< 0.001
Empathy → willingness to accept	0.147	0.025	4.523	< 0.001
Social Norms → willingness to accept	0.106	0.025	3.550	< 0.001
Satisfaction → willingness to accept	0.179	0.034	5.097	< 0.001

β, Standardized path coefficient; S.E, Standard Error. All path coefficients were statistically significant at *p* < 0.001.

## 4 Discussion

### 4.1 Overview of key findings

This study employed a rigorous structural equation modeling (SEM) approach to delineate the determinants of orthodontic treatment acceptance among a representative sample of university students in Liaoning Province, Northeast China (1, 22). Our analysis of 1,299 respondents revealed a notable homogeneity in perceptions across major demographic strata, suggesting that within this specific population segment, factors such as gender, household registration (hukou), and regional background may be less influential than shared psychosocial and cognitive evaluations in shaping orthodontic intentions. The core analytical model demonstrated excellent fit, supporting the hypothesized pathways (28, 35). The findings illuminate a dual-channel mechanism: the four fundamental dimensions of perceived service quality—reliability, responsiveness, assurance, and empathy—directly enhance anticipated treatment satisfaction. Concurrently, this anticipated satisfaction, the direct appraisal of the same service quality facets (particularly reliability, responsiveness, and assurance), and the perceived pressure of social norms collectively and directly foster a greater willingness to accept treatment. This model moves beyond identifying isolated correlates to mapping the cognitive-affective architecture underlying pre-treatment decision-making in a non-clinical, young adult cohort.

### 4.2 Interpretation of findings in the context of existing literature

The pronounced influence of assurance (technical competence and trustworthiness) and reliability (consistency and dependability) on both satisfaction and willingness aligns robustly with the core tenets of healthcare service evaluation models and prior dental research (18, 26, 27). These dimensions address fundamental patient concerns about safety, efficacy, and predictable outcomes, which are paramount in a healthcare context involving irreversible procedures and long-term commitments (10, 29). Our finding that assurance was the strongest predictor of satisfaction (β = 0.230) underscores that for university students, the perceived expertise and credibility of the orthodontist form the bedrock of positive treatment expectations, a concern that likely transcends cultural contexts (17, 25).

The significant roles of responsiveness and empathy resonate with a growing body of literature emphasizing patient-centered communication and the relational aspect of care as critical components of healthcare quality (24). In orthodontics, where treatment is prolonged and involves regular adjustments, the clinic's willingness to respond to concerns and provide emotional support (empathy) is not merely a courtesy but a functional component of managing treatment-related anxiety and sustaining patient engagement (9, 14). The direct effect of responsiveness on willingness (β = 0.199) suggests that prompt and attentive service is perceived by students as reducing logistical and psychological burdens, thereby lowering a practical barrier to initiating treatment. The inclusion and validation of social norms as a significant, albeit modest, direct predictor (β = 0.106) provides crucial empirical support for the application of psychosocial theories like the Theory of Planned Behavior (TPB) in orthodontic decision-making research (12, 16). This confirms that behavioral intention is shaped not only by personal attitudes (here, largely formed by service quality perceptions and derived satisfaction) but also by the perceived expectations of one's social referent group (7). In the collective social milieu of a Chinese university, where peer influence is potent, this normative component adds a necessary layer of social contextualization to the decision-making model, bridging micro-level perceptions with macro-level social influences (13, 20). The absence of significant demographic differentials, while contrasting with studies highlighting socioeconomic barriers (8, 11), may reflect the unique characteristics of our sample. University students, despite varied backgrounds, occupy a shared social ecosystem with relatively homogeneous access to information, comparable lifestyle rhythms, and similar future orientations. This environment may foster a convergence in health service expectations and aesthetic values, temporarily attenuating the effects of traditional demographic disparities on perceptions and intentions (3, 7). However, this finding should not be misinterpreted; demographic and economic factors undoubtedly re-assert their influence at the stage of translating intention into actual behavior and treatment affordability (4).

A theoretically rich and somewhat nuanced finding is the dual pathway through which service quality dimensions operate. Their effects are not fully mediated by anticipated satisfaction; significant direct paths to willingness persist. This suggests that cognitive evaluations of service attributes have instrumental, utilitarian value independent of the positive affective state they generate (23). For example, high perceived reliability may directly mitigate fears of treatment failure or unexpected complications, thus reducing perceived risk—a key barrier in health behavior models (15). Similarly, assurance may directly bolster confidence in overcoming treatment challenges. This indicates that individuals engage in a more complex calculus, weighing both the emotional payoff (satisfaction) and the pragmatic utility (risk reduction, confidence building) derived from their perceptions of service quality. This complexity underscores the advantage of SEM in capturing these parallel processes over traditional regression models that might only test for mediation (21, 34). Furthermore, the differential strength of the path coefficients offers granular insights for intervention. The dominance of assurance in driving satisfaction highlights the non-negotiable importance of communicating professional expertise. Conversely, the strong direct effect of responsiveness on willingness suggests that operational efficiency and communicative accessibility are powerful independent motivators for action. This implies that a clinic perceived as highly skilled but unresponsive might generate satisfaction in theory but fail to motivate initial contact, whereas a clinic seen as very responsive and adequately assuring might successfully convert intention.

This study makes a substantive theoretical contribution by systematically integrating the multi-dimensional SERVQUAL framework from service marketing (26, 27) with the psychosocial constructs of the TPB (12, 16) within a cohesive SEM. This integration responds to calls for more comprehensive models in health behavior research that account for both the attributes of the healthcare “product” and the psychological processes of the individual within their social context (15, 27). Our model effectively positions service quality perceptions as formative of the “attitudinal” component in the TPB, which then, alongside the “subjective norm” component, predicts intention. This provides a concrete mechanism for how external information about a service translates into internal drivers of behavior. Methodologically, the application of SEM, following the established two-step approach of assessing measurement then structural models, represents a robust advancement in this field of inquiry (22, 34). By explicitly modeling latent constructs and accounting for measurement error, our analysis provides more reliable and valid estimates of the relationships among these complex, abstract variables than would be possible with simpler techniques (21, 33). The confirmation of good model fit using multiple indices (χ^2^/df, CFI, TLI, RMSEA) adhering to contemporary standards (28, 35) lends strong empirical support to the proposed theoretical structure. It validates the conceptualization of treatment acceptance as a process mediated by cognitive-affective states and moderated by social context, moving the field from a factor-listing phase toward a theory-testing and mechanism-elucidating phase.

Acknowledging the limitations of this research is essential for interpreting its findings and guiding future inquiry. First, the cross-sectional design, while efficient for mapping associations, inherently limits causal inference (34). Although the model directions are grounded in theory, longitudinal or experimental studies are required to robustly establish whether changes in service quality perceptions cause subsequent changes in satisfaction and willingness. Second, common method bias is a potential concern as all data were self-reported via a single questionnaire (33). While we employed psychometrically sound scales and anonymous administration to mitigate this, future research could benefit from multi-trait, multi-method approaches, such as combining surveys with analysis of online reviews or experimental vignette studies. Third, our dependent variable was behavioral intention (willingness), not observed behavior. The intention-behavior gap is a well-documented phenomenon across health domains (12, 34). Factors such as actual financial constraints, access to care, and acute life events may intervene between a student's expressed willingness and their actual pursuit of treatment. Prospective studies that track participants from intention to consultation and treatment commencement are crucial. Fourth, the generalizability of our findings is constrained by the specific context—university students in one province of Northeast China. Cultural values regarding aesthetics, familial influence on healthcare decisions, and the structure of the healthcare system can vary widely (13, 19). Replicating this model in other cultural and healthcare delivery contexts (e.g., different regions of China, countries with nationalized vs. private dental care) is necessary to test its universality and identify cultural moderators. Fifth, while our model explained a significant portion of the variance, it is not exhaustive. Other potentially influential variables not measured here include specific detailed cost/insurance perceptions, individual personality traits (e.g., pain anxiety, vanity), objective orthodontic treatment need, and past traumatic dental experiences. Incorporating these variables in future models could enhance explanatory power (36). Finally, the sampling, while methodologically sound using PPS and cluster techniques (31, 32), focused on students with prior awareness of orthodontics. This excludes those completely unaware, whose decision-making process would begin at an earlier stage of knowledge acquisition, potentially involving different factors.

## 5 Conclusion

In conclusion, this study advances the understanding of orthodontic treatment acceptance by proposing and validating an integrated theoretical model that synthesizes service quality evaluation with psychosocial behavioral theory. It demonstrates that for Chinese university students, the journey toward accepting orthodontic care is guided more by shared evaluations of how care is delivered and perceived social cues than by traditional demographic categories. The dual pathway of service quality—affecting both the heart (satisfaction) and the mind (pragmatic assessment)—reveals the sophisticated nature of healthcare decision-making among educated young adults. For clinical practitioners and practice managers, the implications are strategic. Beyond investing in advanced technology, marketing should consciously communicate assurance (e.g., credentials, case showcases) and reliability (e.g., clear treatment plans, outcome guarantees). Operational policies must prioritize responsiveness (e.g., efficient scheduling, prompt query handling) and staff training must foster genuine empathy. Clinics should also consider how to positively shape social norms, perhaps through satisfied young adult patient testimonials or partnerships with university health centers. For public health policymakers and university health advocates, the findings suggest that campaigns to improve orthodontic uptake should address these quality dimensions in informational materials. Interventions could simulate positive service experiences (e.g., consultation workshops) and leverage peer networks to create supportive social norms around seeking orthodontic care when needed. By employing a sophisticated SEM methodology and integrating diverse theoretical lenses, this research provides a replicable framework for exploring health service acceptance. It underscores that in an era of informed patients and competitive healthcare markets, understanding the nuanced psychology of choice is as critical as advancing clinical technique for improving public oral health outcomes.

## Data Availability

The original contributions presented in the study are included in the article/Supplementary material, further inquiries can be directed to the corresponding authors.
