# Correction: Determinants of orthodontic treatment acceptance among university students in Northern China: a structural equation modeling approach

**DOI:** 10.3389/fpubh.2026.1900808

**Published:** 2026-06-17

**Authors:** Peng Zhang, Zhiliang Chu, Jiarui Li, Yue Chai, Renbin Han, Li Dong

**Affiliations:** 1 School of Stomatology, Jinzhou Medical University, Jinzhou, Liaoning, China; 2 School of Health Management, Jinzhou Medical University, Jinzhou, Liaoning, China; 3 Innovation and Entrepreneurship Center, Jinzhou Medical University, Jinzhou, Liaoning, China

**Keywords:** China, orthodontics, service quality, structural equation modeling, treatment acceptance, university students

Author Peng Zhang was erroneously assigned as corresponding author. The correct corresponding author is Li Dong only.

The original version of this article has been updated.

